# Characterization of e-cigarette users according to device type, use behaviors, and self-reported health outcomes: Findings from the EMIT study

**DOI:** 10.18332/tid/174710

**Published:** 2023-12-05

**Authors:** Anna Tillery, Angela Aherrera, Rui Chen, Joyce J. Y. Lin, Mina Tehrani, Donia Moustafa, Jana Mihalic, Ana Navas-Acien, Ana M. Rule

**Affiliations:** 1 Johns Hopkins Bloomberg School of Public Health, Johns Hopkins University, Baltimore, United States; 2 Mailman School of Public Health, Columbia University, New York, United States

**Keywords:** ENDS, electronic cigarettes, behaviors, population studies, demographic classification

## Abstract

**INTRODUCTION:**

Electronic cigarettes (e-cigarettes) rapidly evolved from large modifiable (MOD) devices, to small and affordable ‘POD’ devices. Detailed information on user demographics and preferences according to device type, which can inform potential chemical exposure and policy recommendations, is currently limited. The goal of this study is to describe user demographics, use behaviors and preferences, as well as self-reported health outcomes according to the e-cigarette device type used.

**METHODS:**

From April 2019 to March 2020, 91 participants from Maryland (18 MOD users, 26 POD users, 16 dual users (use of both combustible and e-cigarettes), and 31 non-users (never e-cigarette users and never smokers or >6 months former use) were recruited. A comprehensive questionnaire collected sociodemographic characteristics, e-cigarette/tobacco use behaviors, self-reported health outcomes, device characteristics and preferences. Chi-squared tests for categorical variables, ANOVA for continuous variables, qualitative thematic analysis, linear and logistic regressions were used to assess relationships between variables and groups.

**RESULTS:**

POD users were younger (average 22.5 years) than MOD users (30.8 years) or dual users (34.3 years) (p<0.001). MOD users reported more puffs per day (mean ± SD: 373 ± 125 puffs) compared to POD users (123.0 ± 172.5). E-cigarette users who were former smokers used 1.16 mg/mL lower nicotine concentrations compared to lifetime exclusive e-cigarette users (p=0.03) in linear models. Exclusive POD users self-reported more coughing than exclusive MOD or dual users (p=0.02). E-cigarette users reported more shortness of breath, headaches, and fatigue from their e-cigarette use compared to non-users.

**CONCLUSIONS:**

We found significant differences between user demographics, e-cigarette preferences, device characteristics, and use behaviors by user group. This information can help explain exposure to chemicals from e-cigarettes, including compounds with known toxic effects (e.g. metals, formaldehyde), and help inform the design of prevention and intervention strategies and policy decisions.

## INTRODUCTION

The use of electronic cigarettes (e-cigarettes) has steadily gained popularity among young adults due to heavy marketing that includes appealing flavors and concealability^1,2^. As of 2022, more than 1 in 10 middle and high school students (3.08 million), and 4.5% of the adults aged ≥18 years (11.1 million) reported currently using e-cigarettes in 2021^3,4^. While e-cigarette devices have evolved over the years, all share three main components: a battery, an atomizer, and e-liquid. First generation e-cigarettes, known as ‘cig-a-likes’, mimic combustible cigarettes with a non-refillable e-liquid cartridge^5^, while second (e-pens) and third [modifiable e-cigarettes (MODs)] generation devices are open systems with refillable tanks; MODs allow customization of device characteristics such as voltage, wattage, temperature, and type of heating coils. Fourth generation devices (PODs), similar to ‘cig-a-likes’, are closed design systems with replaceable cartridges that contain the e-liquid. Popularized by the brand JUUL, PODs resemble USB flash drives with USB charging ports and often contain high nicotine concentrations (50 mg/mL)^5^. The newest (5th) generation of e-cigarettes – disposable PODs (d-PODs) introduced in 2021 – are similar in style to PODs but are disposable (‘vape-and-throw’) that offer up to 7000 puffs. Although the SARS-CoV-2 pandemic may have changed users’ access to e-cigarettes while living at home, a recent study found no significant difference between youth use before and during the pandemic^1^ , citing PODs and d-PODs as commonly used devices^6^.

Since e-cigarettes were first introduced, device type purchases and preferences have changed over time^7,8^. Important differences in device construction between generations of devices alongside user preferences, including preferred nicotine concentration, may lead to significant differences in chemical exposures and impact health outcomes^9-13^. Moreover, differences in reported health outcomes may be associated with e-cigarette use status [i.e. exclusive vs dual (uses e-cigarettes and smokes combustible cigarettes), vs non-user]^12^. Understanding e-cigarette use and whether use behaviors are associated with certain user demographics is important to recognize who may be at higher risk for exposures to chemicals that have been found in e-cigarette aerosols, such as contaminant metals and aldehydes^9,13^. Lastly, while nationally representative studies such as the Population Assessment of Tobacco and Health (PATH) study and the National Health Interview Survey (NHIS) ask questions pertaining to e-cigarette use, nuanced questions regarding device characteristics (i.e. power, heating coil) and use behaviors (i.e. coil change/month, amount of e-liquid (mL) or number of cartridges used per week) are pertinent factors to consider when understanding e-cigarette use and potential chemical exposure. Compared to our previous analysis, this study evaluates more recent types of devices that have come to market and grown in popularity, including POD devices with relatively higher nicotine concentration, which have been responsible for the vaping epidemic among youth and young adults^14^. The purpose of this study was to evaluate e-cigarette use behaviors, device characteristics, and self-reported health outcomes by comparing exclusive e-cigarette users to dual users, as well as comparing the type of e-cigarette devices (POD, MOD) used among a cohort in Maryland.

## METHODS

### Study population and recruitment

Participants were recruited into one of four categories based on e-cigarette and tobacco use: non-users, exclusive POD users, exclusive MOD users, and dual users of combustible and electronic cigarettes. Recruitment was conducted through advertisements and flyers posted in universities and e-cigarette (vape) shops and conventions between April 2018 and March 2020 in Maryland, USA. Participants were residents of Maryland, aged ≥18 years, and not pregnant at the time of recruitment. Exclusive e-cigarette users were defined as never cigarette smokers or former smokers who had quit at least 6 months before enrollment, vaped regularly for at least 6 weeks, and did not live with a traditional smoker. Dual users were defined as using both e-cigarettes and combustible tobacco products at least 6 weeks before enrollment, using e-cigarette products and smoking combustible tobacco products most days of the week. Non-users were defined as non-combustible tobacco product users and non-e-cigarette users, or former users who quit at least 6 months prior to enrollment. To aid in comparability between groups, participants were asked to refer a matching friend/family member; non-users were matched to e-cigarette users according to age (within 5 years), sex, and race. Disposable PODs were not commonly used during the recruitment period, so none of our participants was a d-POD user. The study protocol was approved by the Institutional Review Board at Johns Hopkins University (Baltimore, Maryland). All participants provided written informed consent.

### Data collection

After confirming eligibility, participants who use e-cigarettes (POD, MOD, and dual users) were asked to carry out their normal vaping routine and bring their e-cigarette device to the study visit, which took place at the Johns Hopkins Bloomberg School of Public Health in Baltimore, MD. At the time of their appointment, participants responded to a (120-question) interviewer-based questionnaire addressing sociodemographic characteristics, previous tobacco use, current e-cigarette use [including e-liquid consumed/week, preferred voltage, nicotine concentration, puffs/day, time to first vape in the morning (minutes), last coil change (days), coil change/month, time to finish 1 POD (days)] etc. Nicotine concentration was converted from mg/mL to percentage when applicable; percentages were converted as 1/10th reported mg/mL nicotine as is standard^15^. All participants were asked about their overall health and whether they experienced sensory (i.e. irritated eyes, runny nose, sore throat, headache) and respiratory symptoms (i.e. coughing in the morning or at night, feeling short of breath, wheezing in the chest) at all during the last 4 weeks. E-cigarette users (i.e. exclusive users and dual users) were also asked about their overall health since using e-cigarettes (positive and/or negative health changes).

### Statistical analysis

Demographic characteristics, e-cigarette use behaviors, and self-reported health outcomes were compared across groups using chi-squared tests for categorical variables and analysis of variance (ANOVA) for continuous variables. We ran linear regression models to compare the mean differences in e-cigarette use behaviors [e-liquid nicotine concentration (%), puffs per day, power, last coil change, coil changes per month, e-liquid consumed per week, time to finish one POD], by demographic characteristics, adjusting for age, sex, race, education level and former smoking status. We also ran logistic regression models to analyze time to first vape from waking in the morning [≤15 minutes (reference group) or >15 minutes after waking] by demographic characteristics, adjusting for the same covariates. Age, sex, and race were covariates included in the model due to their associations with tobacco use in the previous literature^8,14,16^. We identified and grouped emergent themes in reported health outcomes of e-cigarette users for qualitative thematic analysis. Statistical analysis was conducted in RStudio Version 1.3.1093 (RStudio, PBC). The statistical significance level was set at α=0.05.

## RESULTS

### Demographic characteristics

Ninety-one participants (31 non-users, 18 MOD users, 26 POD users, and 15 dual users) were recruited (Table 1). Participants’ mean age was 28.1 years (SD=9.59) and most participants were male (57%) and White (56%). POD users were younger (mean age: 22.5 years) (p<0.001), more likely to be full-time students (77%) (p<0.001), and never smokers (69%) (p<0.001) compared to other groups, while most MOD users were former smokers (88%). According to race, most MOD (78%), POD (62%), and dual (56%) users were White. All dual users reported using MOD devices as their primary e-cigarette device; 17% reported using JUUL/POD devices in addition to their primary MOD device.

**Table 1:** Sociodemographic characteristics of participants by tobacco use category from the EMIT cross-sectional study, 2018–2020 (N=91)

*Characteristics*	*n*	*Total (N= 91) %*	*Exclusive e-cigarette users*		*Dual users (N=16) %*	*Non-users (N=31) %*	p*
			*POD users (N=26) %*	*MOD users (N=18) %*			
**Age** (years), mean (SD)		28.1 (9.59)	22.46 (5.17)	30.83 (8.68)	34.25 (11.62)	28.03 (9.54)	**<0.001**
**Gender**							
Male	52	57	58	78	69	39	**0.04**
Female	39	43	42	22	31	61	
**Education level**							
≤High school	17	19	12	23	0	11	0.4
>High school	74	81	88	77	100	89	
**Race**							
White	56	38	62	78	56	55	0.4
Non-White	35	62	38	22	44	45	
**Ethnicity**							
Non-Hispanic	63	11	77	88	88	100	0.3
Hispanic	8	89	23	12	12	0	
Unanswered	20						
**Employment status**							
Employed	42	49	73	33	56	48	0.06
Student	44	48	77	22	19	55	**<0.001**
Smoking status**							
Never smoker	47	64	69	12	-	90	**<0.001**
Ever smoker	26	36	31	88	-	10	

* ANOVA and chi-squared for continuous and categorical variables, respectively.

** Never cigarette smokers (Never smoker) or former smokers who had quit at least 6 months before enrollment (ever smokers).

### E-cigarette use behaviors and device characteristics

More MOD (89%) and dual users (93%) indicated that they used their device every day compared to POD users (73%) (Table 2) (p=0.2). MOD users, on average, started vaping in 2015 compared to dual users who started in 2016 and POD users in 2017 (p=0.05). MOD users reported the highest average number of puffs per day [mean (SD): 373 (125); median (range): 100 (10–2160) puffs] compared to POD users [123 (72.5); 70 (5–300) puffs) and dual users (107 (102); 100 (12–400) puffs]. Most MOD users (59%) start vaping ≤15 minutes from waking up in the morning compared to both POD (27%) and dual users (47%) (p=0.10). MOD and dual users preferred fruit flavors versus POD users who preferred menthol/mint flavors (p<0.001). Most MOD users (67%) bought their devices at vape shops while POD users bought them at gas stations or online (85%, p<0.001); dual users were evenly split between buying at vape shops and gas stations.

**Table 2:** Self-reported e-cigarette use behaviors stratified by type of e-cigarette user (POD, MOD, dual user) from the EMIT cross-sectional study, 2018–2020 (N=60)

*E-cigarette use behaviors*	*All users (N=60) %*	*POD users (N=26) %*	*MOD users (N=18) %*	*Dual users (N=16) %*	*p*
**Year participant began vaping, median**	2017	2018	2016	2018	0.05
**Year participant started using current device, median**	2018	2018	2018	2018	>0.9
**Location of e-cigarette purchase**					
Vape shop	37	15	67	40	**<0.001**
Online	19	15	28	13	
Other	44	70	5	47	
**How often device is used**					
Everyday	83	73	89	93	0.2
Some days	17	27	11	7	
**Time to first vape** (min)					
≤15	41	27	59	47	0.1
>15	59	73	41	53	
**Nicotine concentration** (%), median (range)	5 (1.2–6)	5 (1.2–6)	4 (2.5–6)	5 (1.5–6)	0.9
**Puffs per day**, median (range)	80 (5–2160)	70 (5–300)	100 (10–2160)	100 (12–400)	0.4
**Device power** (W), median (range)	39 (14.4–100)	-	37 (14.4–100)	38 (26–50)	0.7
**Preferred flavor category**					
Fruit	41	27	58	55	**<0.001**
Menthol/mint	39	62	0	27	
Candy/dessert	12	8	33	0	
Tobacco	8	3	9	18	
**How long to finish 1 Pod** (days)					
1	31	29	-	38	0.6
3–7	53	50	-	62	
>7	16	21	-	0	
**E-liquid consumed per week** (mL), median (range)	18.75 (1–220)	-	18.75 (2–220)	8 (1–100)	0.8
**Coil change per month**, median (range)	1.5 (1–3)	-	1.5 (1–3)	1.5 (1–3)	0.9
**Days since last coil change**, median (range)	7 (1–152)	-	7 (1–152)	25.5 (2–60)	0.4

For MOD and dual users, the average consumption of e-liquid per week was 44.3 mL (SD=56.1) and the median consumption was 18.75 mL (range: 1–220). Among POD users, the majority of participants finished 1 pod in <1 week. The overall average nicotine concentration reported was 4.51% (SD=1.28, range: 0–6) (Table 2). Former smoking status was negatively associated with vaping and nicotine percentages (p<0.001) (Table 3). Although not statistically significant, older participants consumed more e-liquid per week; women consumed less e-liquid per week than men. Lastly, those with a greater than a high school education level reported using their device at lower power and lower nicotine concentrations than those with a high school education level or less.

**Table 3:** Mean difference (95% CI) in e-cigarette use by demographic characteristics analyzed using linear and logistic regression from the EMIT cross-sectional study, 2018–2020 (N=60)

*Characteristics*	*Nicotine (mg/mL) (95% CI)*	*Puffs per day (95% CI)*	Time to first vape** (95% CI)	*Power (Watts) (95% CI)*	*Last coil change (days) (95% CI)*	*Coil change per month (95% CI)*	*E-liquid consumed per week (mL) (95% CI)*	Time to finish 1 POD (days)* (95% CI)
**Age** (years)	0.043 (-0.023–0.109)	9.48 (-12.1–31.0)	0.967 (0.869–1.07)	0.069 (-1.84–1.98)	0.344 (-2.47–3.15)	0.019 (-0.041–0.079)	3.46 (-0.459–7.38)	0.925 (0.794–1.05)
p	0.216	0.395	0.265	0.945	0.814	0.545	0.107	0.955
**Gender**								
Male (Ref.)	0.00	0.00	0.00	0.00	0.00	0.00	0.00	0.00
Female	0.400 (-0.429–1.22)	-63.1 (-332–206)	0.505 (0.018–2.36)	-10.4 (-43.1–22.4)	28.5 (-18.0–74.9)	-0.332 (-1.33–0.668)	-45.4 (-110–19.4)	0.505 (0.108–2.36)
p	0.350	0.649	0.375	0.578	0.252	0.527	0.193	0.791
**Education level**								
≤ HS (Ref.)	**0.00**	0.00	0.00	**0.00**	0.00	0.00	0.00	0.00
> HS	**-0.415 (-1.47–0.638)**	-100 (-442–242)	3.34 (0.515–23.4)	**-43.2 (-81.7–23.6)**	9.38 (-47.9–66.6)	0.582 (-0.651–1.81)	-28.4 (-108–51.5)	3.34 (0.515–23.4)
p	**0.445**	0.569	0.203	**0.053**	0.753	0.372	0.499	0.484
**Race**								
Non-White (Ref.)	0.00	0.00	0.00	0.00	0.00	0.00	0.00	0.00
White	0.009 (-0.833–0.851)	-28.2 (-301–245)	0.673 (0.128–3.13)	-7.17 (-37.9–23.6)	-0.324 (-44.1–43.5)	0.448 (-0.495–1.39)	27.7 (-33.4–88.8)	0.673 (0.129–3.13)
p	0.983	0.841	0.619	0.658	0.988	0.369	0.390	0.130
**Previous smoker**								
No (Ref.)	**0.00**	0.00	0.00	0.00	0.00	0.00	0.00	0.00
Yes	**-1.16 (-2.17 – -0.152)**	152 (-175–480)	0.482 (0.074–3.01)	27.1 (-14.0–68.1)	5.94 (-59.5–71.4)	0.980 (-0.430–2.39)	33.3 (-58.0–125)	0.482 (0.074–3.01)
p	**0.030**	0.368	0.430	0.226	0.862	0.196	0.488	0.536

Models adjusted for: age, sex, race (White/Non-White), education (HS or less/Greater than HS), and former smoking status.

* Excludes MOD users.

** Logistic regression, adjusted odds ratios (AORs) presented; age, sex, and race were covariates included in the model [>15 min, ≤15 min (Ref.)].

### Self-reported health outcomes

**Figure 1:**
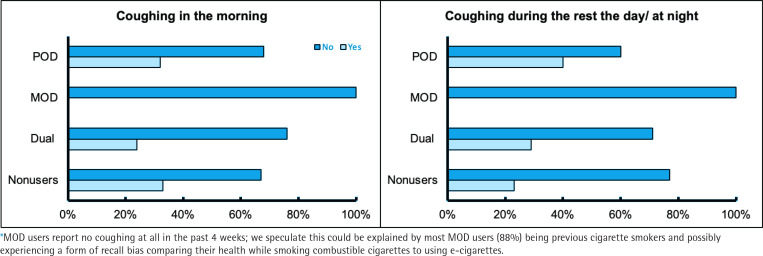
Self-reported coughing (%) in the morning (p=0.028), during the day, and rest of the night according (p=0.015) to user group (POD user, MOD user, dual user, and non-user) (n = 91) from the EMIT cross-sectional study, 2018–2020*

No users reported any pre-existing health conditions diagnosed by a medical professional. Reports of coughing in the morning were significantly different (p=0.028) between user groups (MOD 0%, POD 32%, dual 24%, and non-users 33%), as well as significant differences (p=0.015) were reported for coughing during the day or at night for MOD (0%), POD (40%), dual (29%), and non-users (23%) (Figure 1). Upon excluding MOD users in the analysis of coughing in the morning as well as coughing during the rest of the day or at night, no significant differences were found between POD users, dual users, and non-users. No significant associations were found between self-report health outcomes and demographic characteristics.

Among both exclusive e-cigarette users and dual users, 37% reported positive health effects associated with the use of e-cigarettes, while 31% reported negative health effects since they began vaping (Supplementary file Figure 1). Fifteen (34%) exclusive e-cigarette users reported positive health effects associated with e-cigarette use, including better breathing (n=7), better cardiovascular effects (n=3), helps with anxiety (n=2), increased energy (n=2), less exposure to chemicals and secondhand smoke (n=2), better skin (n=1), and better sleep (n=1). Seven (44%) dual users reported positive health effects associated with e-cigarette use, including less coughing compared to tobacco cigarettes (n=4), better breathing and lung capacity (n=3), less throat soreness (n=3), and helps with relaxing (n=1). Eleven (25%) exclusive e-cigarette users reported negative health effects associated with e-cigarette use, including having respiratory outcomes (n=5), issues with stamina and reduced energy (n=4), headache (n=2), nausea (n=2), a sore throat (n=1), and dehydration during use (n=1). Lastly, six dual users (37%) reported negative health effects associated with e-cigarette us, including having respiratory outcomes (n=5), headache (n=3), mental health effects (n=3), and throat pain (n=2).

## DISCUSSION

In our convenience sample of 91 participants from Maryland, recruited between 2018 and 2020, most of our study participants were White (56%), non-Hispanic (63%), men (52%) and had greater than a high school education level (74%). This is consistent with data from the nationally representative Population Assessment of Tobacco and Health (PATH) study (Wave 5, December 2018 to November 2019), where exclusive use of e-cigarettes was more prevalent among non-Hispanic Whites and men compared to non-Hispanic Blacks, Hispanics, and women^17,18^. POD user demographics in our study are also consistent with previous literature^14^, with POD users being the youngest group, majority students, and never smokers compared to MOD and dual users. Zare et al.^8^, found that user demographics vary by e-cigarette device preferences, with women more likely to choose disposable and POD devices than men. Our study is unique as it not only analyzes detailed questions about e-cigarette use behaviors, not typically asked in nationally representative surveys such as PATH and NHIS, but also characterizes behaviors and preferences according to the different types of devices used, which can lead to distinct chemical exposures. We found significant differences in use behaviors between POD, MOD, and dual user groups. POD users, on average, used their devices less frequently than MOD or dual users, and were more likely to be never smokers than MOD users, which is consistent with studies by Mantey et al.^19^ and Soneji et al.^20^, which found that young adults (18–24 years) vaped fewer days per month than older adults (Ñ25 years). We found that POD users were significantly younger than MOD users and less likely to be former smokers. POD users’ less frequent use, compared to MOD users, is likely attributed to the higher nicotine content typically found in PODs, thus requiring fewer ‘hits’^21^. Our findings are consistent with those from a systematic review by Zare et. al.^16^, that found former smokers preferred open and modifiable e-cigarette systems to closed systems, and that former smokers have past reliance on frequent ‘hits’.

We found that MOD and dual users significantly preferred fruit flavor categories compared to POD users, who preferred mint and menthol flavors. Under pressure from the FDA, JUUL suspended sales of most of its flavored products in October 2019, (including mango and mint), which was enforced by the FDA by January 2020, leaving tobacco and menthol in the market^6^, which could have influenced POD users’ responses. The systematic review by Zare et al.^16^ found that vapers prefer flavored e-cigarette liquids, with the two most common flavor preferences for all e-cigarette users being fruity and mint/menthol^16^. Additionally, they found that sweet flavor preference was stronger in young adults and women. We found that POD users were more likely to purchase their devices/pods at gas stations or online compared to MOD users who buy at vape shops. This could indicate that convenience of access to POD devices is a factor for use in this population.

We also found differences in demographic characteristics and use behaviors. We found that on average, women vaped less e-liquid volume per week than men, consistent with previous literature^14,22^. We found that older users vaped more e-liquid per week, which is inconsistent with our previous study on demographics and vaping behaviors that found no significant relationship between age and e-liquid used, although this study was conducted prior to the emergence of PODs in the market^14^. Indeed, our most recent findings are likely explained by older participants’ preference for MOD devices that use e-liquids of lower nicotine content. POD devices, which are more popular among younger users, contain higher nicotine, which would require less consumption of e-liquid to achieve the same nicotine dose^23^. We also found that higher education level was associated with lower nicotine, lower power, and more frequent coil change. In a 2020 study of race, education level, and e-cigarette use, Assari et al. ^24^ found an inverse association between education level and e-cigarette use among Whites, and a positive association among non-Whites; the authors surmise this positive association may be explained by lower general health literacy and/or lower perceived harm of e-cigarettes in highly educated non-Whites. This may also be explained by tobacco industry marketing strategies that may specifically target people of color and of lower socioeconomic status (SES)^25^. A 2022 review, by Addo Ntim et al.^25^ on prevalence and use, found higher advertisement exposure among individuals of lower SES, which was defined by a number of factors, including user’s education level; individuals with lower SES were more likely to report using e-cigarettes because people in the media or other public figures used them compared to those in higher SES groups.

Our study found that previous smokers used lower nicotine concentrations than vapers who have never smoked cigarettes before. These findings are inconsistent with the findings by Zare et al.^16^, who found that former smokers preferred higher nicotine content compared to non-smokers. This discrepancy could be explained by most previous smokers in our study using MOD devices rather than high nicotine-containing POD devices and/or by the average age differences between the Zare et al.^16^ study (52 years) and ours (29 years).

A recent study by Vargas-Rivera et al.^26^ found that demographics and vaping preferences are associated with e-cigarette use behaviors. Users who prefer JUUL POD devices, have been shown to vape more frequently when using their favorite flavor category^26^. In addition, place of purchase and availability of e-cigarette devices and flavors have been shown to alter purchasing behavior of users^26^. During SARS-CoV-2 lockdowns and online schooling, youth e-cigarette users reported changing place of purchase to online retailers, bypassing age restrictions^1^. Additionally, policies banning certain JUUL POD flavors impacted use behaviors; as certain JUUL flavors disappeared from the market, users switched to disposable e-cigarettes which can still be found in a variety of flavors and are readily available through online retailers^5^.

Although we found no differences in most sensory and respiratory symptoms, we found significant differences in self-reported coughing in the morning as well as coughing during the rest of the day and night among the different user groups; MOD users reported no coughing (0%) compared to POD, dual, and non-users who reported coughing at similar rates (about 30%). We are uncertain as to why there is a discrepancy with MOD users reporting no coughing at all in the past 4 weeks; we speculate this could be explained by most MOD users being previous cigarette smokers (88%) and possibly experiencing a form of recall bias comparing their health while smoking combustible cigarettes to using e-cigarettes. Although we found no significant differences between self-reported health outcomes and demographic characteristics, former studies have found that demographic characteristics are associated with health effects from e-cigarette use, with negative health effects such as headache and coughing being the most reported^27^. This is important as it could mean the study has low statistical power or perhaps that this self-reported health effect may not be of clinical significance. It could also possibly indicate that there are no substantial short-term health effects of e-cigarettes with respect to self-reported coughing. The differences between user groups and reported health outcomes could be explained by the significant differences between e-cigarette users’ preferences and device types. The most common reported negative health outcomes found in our study, align with research by Hua et al.^27^ conducted with self-reported data on e-cigarette users. Additionally, many of the positive health effects reported by e-cigarette users could be explained by nicotine addiction as they resemble the effects of nicotine withdrawal and alleviation during nicotine use^28^.

Approximately one-third of POD users reported coughing in the morning (32%) and during the day and at night (40%). As these POD users who indicated ‘yes’ were young (mean age: 23 years), mostly never smokers (80%), and JUUL users (100%), such information indicates POD devices’ potential impact on young adults’ respiratory health, distinct from using MOD devices or dual use. Multiple studies have found that the most common self-reported health effects from e-cigarette use is coughing, but these studies did not differentiate by user device type^29,30^. Whether these reported symptoms are related to pre-clinical function or tissue level alterations in the lungs is unknown and should be investigated.

### Limitations

This study has several limitations. Our recruitment was interrupted first by the EVALI epidemic in 2019 and then by the SARS-CoV-2 pandemic, and thus our small sample size affected statistical power. Despite the small sample size, our study was able to capture more detailed information about e-cigarette use, preferences, and behaviors than other national tobacco surveys, which can better inform potential chemical exposures unique to the type of user and device used. Our study could also be affected by selection bias due to convenience sampling. For example, e-cigarette use behaviors are based on self-report, and it is possible that participants could have displayed recall or social desirability bias. As we only recruited participants aged ≥18 years, we are missing an important population of e-cigarette users, particularly among youth of middle school and high school age. Moreover, although we employed the criterion of having exclusive e-cigarette users and non-users who were former smokers to have quit a minimum of 6 months in this study as well as in a previous study^14^, to serve as a wash-out period, the effect of combustible tobacco product use might still have remained among these former combustible users. In addition, our study may have limited generalizability to other countries due to differences in available products and tobacco use behaviors. Lastly, disposable PODs were not popularly used at the time of our recruitment and hence our sample does not include disposable e-cigarette users^5^.

Notwithstanding these limitations, our study has several strengths and provides valuable nuanced information on the different device characteristics and use behaviors of e-cigarette users by the preferred device type, which are not asked in nationally representative surveys. Most e-cigarette users were young, White males. Users who were former smokers were more likely to use MOD devices while those who were never smokers were more likely to use POD devices. MOD and POD users had different frequencies of use, with MOD users vaping at a higher number of puffs per day and with more MOD users vaping within 15 minutes of waking compared to POD users. Participants with a higher level of education (greater than high school) vaped at a lower nicotine and wattage, and changed their coils more frequently, compared to participants of lower level of education. E-cigarette users were split on self-reported health outcomes, and many perceive positive health benefits with e-cigarette use. Of note, POD users had the highest reporting of respiratory symptoms (coughing in the morning, during the day, and rest of the night) when using e-cigarettes compared to other e-cigarette users (exclusive MOD users, dual users). Future research should consider the type of device and use behaviors when studying e-cigarettes to inform exposure profiles and better understand potential toxicity and long-term health effects.

## CONCLUSIONS

We found significant differences between user demographics, e-cigarette preferences, device characteristics, and use behaviors by user group. This information can help explain exposure to chemicals from e-cigarettes, including compounds with known toxic effects (e.g. metals, formaldehyde), and help inform the design of prevention and intervention strategies and policy decisions.

## Data Availability

The data supporting this research are available from the authors on reasonable request.
