# Supplementary material for: Characterization of e-cigarette users according to device type, use behaviors, and self-reported health outcomes: Findings from the EMIT study
Source: Tob Induc Dis. 2023 Dec 5;21:159. doi: 10.18332/tid/174710 (PMC10696923; doi:10.18332/tid/174710)
Supplement: Supplementary file 1 [file TID-21-159-s1.pdf]

SUPPLEMENTAL MATERIALS

Supplemental Figure 1. Self-reported positive (n = 22) and negative (n = 18) health outcomes among e-cigarette users (exclusive and Dual e-cigarette users) after starting to use e-cigarettes.

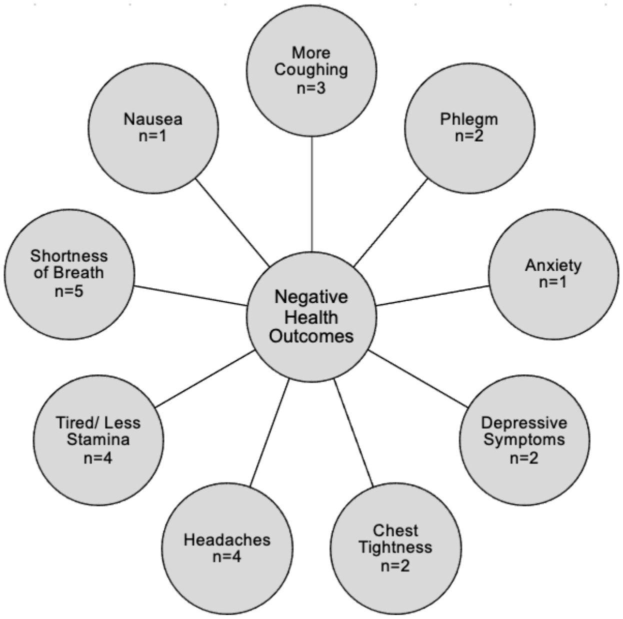

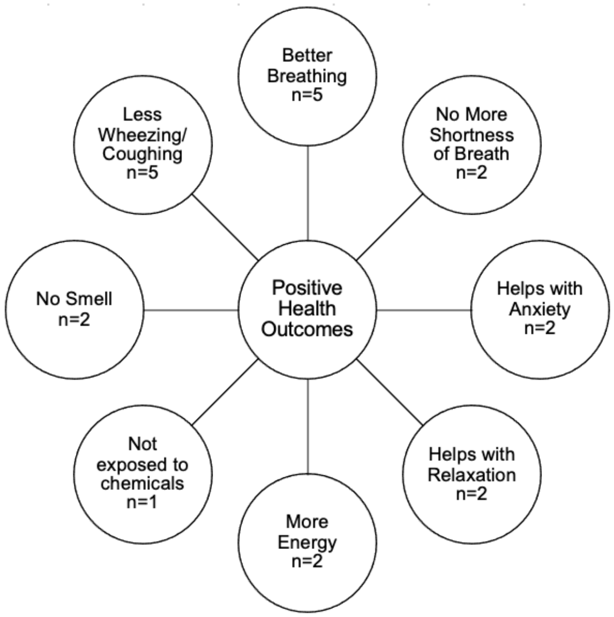

Supplemental Table 1. Self-reported health outcomes questions administered to all participants. All questions were asked with yes/no format; results in affirmative answers (%).

Self-reported health outcomes questions	POD user (n = 26)	MOD user (n = 18)	Dual user (n = 16)	Non-user (n = 31)	*p*-value**
Do you notice any positive health changes after using e-cigarettes?*	33	57	41	-	0.4
Do you notice any negative health changes after using e-cigarettes?*	43	21	35	-	0.4
Has a doctor or other health professional ever told you that you have:					
Asthma	20	12	29	26	0.6
Environmental allergies	28	35	47	29	0.6
Heart disease	0	0	0	3	0.9
Hypertension or high blood pressure	4	6	24	6	0.2
Had a stroke	0	0	0	0	-
Diabetes	0	0	0	0	-
Hypercholesterolemia/high cholesterol	16	6	12	13	0.9
Any other respiratory disease	4	6	12	0	0.2
Do any of the above sensory and respiratory symptoms seem to occur with using e-cigarettes?*	33	29	29	-	0.9
Have you experienced any of the following in the past 4 weeks:					
Red or irritated eyes	28	18	35	16	0.4
Runny nose, sneezing, or nose irritation	64	41	41	45	0.4
Sore or scratchy throat	40	24	18	29	0.4
Headache	50	38	80	62	0.4
Coughing in the morning	32	0	24	32	**0.03**
Coughing during the rest of the day or at night	40	0	29	23	**0.02**

*Questions were only asked to exclusive e-cigarette users and dual users.
**Chi-squared tests

© 2023 Tillery A. et al.
